# From the infant to the geriatric patient—Strategies for inhalation therapy in asthma and chronic obstructive pulmonary disease

**DOI:** 10.1111/crj.13610

**Published:** 2023-04-13

**Authors:** Lars Hagmeyer, Silke van Koningsbruggen‐Rietschel, Sandhya Matthes, Ernst Rietschel, Winfried Randerath

**Affiliations:** ^1^ Institute of Pneumology at the University of Cologne Solingen Germany; ^2^ Clinic for Pneumology and Allergology, Center of Sleep Medicine and Respiratory Care, Bethanien Hospital Solingen Solingen Germany; ^3^ Faculty of Medicine University of Cologne, Pediatric Pulmonology, Allergology and Cystic Fibrosis Center, Children's Hospital Cologne Germany; ^4^ Center for Rare Diseases, Faculty of Medicine University of Cologne, Children's Hospital Cologne Germany

**Keywords:** age, comorbidity, inhalation device, obstructive airway disease

## Abstract

Inhalation therapy represents the standard of care in children, adolescents as well as in young, middle‐aged and geriatric adults with asthma or chronic obstructive pulmonary disease. However, there are only few recommendations for the choice of inhalation devices, which consider both, age‐specific limitations in young and geriatric patients. Transition concepts are lacking. In this narrative review, the available device technologies and the evidence for age‐specific problems are discussed.

Pressurized metered‐dose inhalers may be favoured in patients who fulfill all cognitive, coordinative and manual power requirements. Breath‐actuated metered‐dose inhalers, soft‐mist inhalers or the use of add‐on devices such as spacers, face masks and valved holding chambers may be suitable for patients with mild to moderate impairments of these variables. In these cases, available resources of personal assistance by educated family members or caregivers should be used to allow metered‐dose inhaler therapy. Dry powder inhalers may be reserved for patients with a sufficient peak inspiratory flow and good cognitive and manual abilities. Nebulizers may be indicated in persons who are either unwilling or unable to use handheld inhaler devices. After initiation of a specific inhalation therapy, close monitoring is essential to reduce handling mistakes.

An algorithm is developed that considers age and relevant comorbidities to support the decision‐making process for the choice of an inhaler device.

## Graphical abstract

Inhalation therapy is a highly individualized concept of therapy. Age, maturity and comorbidities have to be considered when choosing a specific inhalation device. Standardized algorithms may support this decision process. A close monitoring is essential as it may detect handling mistakes and the need to modify the device concept. A routine reevaluation of the inhalation concept should be implemented with age, maturation and new occurring comorbidities.

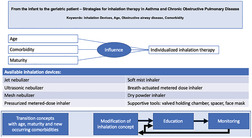

## 1 OBJECTIVES

Obstructive airway diseases are common and may affect infants, children and adolescents as well as young, middle‐aged and geriatric adults. In clinical practice, inhaler usage may be incorrect due to individual limitations in coordination of actuation and inhalation, manual dexterity and power, peak inspiratory flow (PIF) and cognitive status. These limitations may often be challenging in paediatric, geriatric and comorbid patients and show the need to establish individualized recommendations.

It is the aim of this narrative review to give an overview of the available evidence and to derive a proposal for an algorithm for a personalized device therapy in infants, children, adolescents, adults and geriatric patients.

## 2 DATA SOURCE

The PubMed and Cochrane Library databases were selectively searched for current studies, meta‐analyses and guidelines (from 1990 up to July 2022; search restricted to articles published in English language and reporting data from human subjects) on inhalation device use in asthma or chronic obstructive pulmonary disease (COPD).

## 3 STUDY SELECTION

The search terms were ‘asthma’, ‘chronic obstructive pulmonary disease’, ‘inhaler’, ‘inhalation’, ‘device’, ‘nebulizer’, ‘children’, ‘adult’, ‘adolescent’, ‘comorbidity’ and ‘geriatric’. Special attention was paid to the current guidelines and the international Global Initiative for Asthma (GINA) and the Global Initiative for Chronic Obstructive Lung Disease (GOLD) reports. All titles and abstracts were systematically reviewed. Case reports, abstract‐only‐publications, double publications and non‐peer‐reviewed publications were excluded. In case of given relevance of the remaining abstracts, the full‐text publications were analysed in detail and screened for data contributing evidence to the objectives of this review.

## 4 RESULTS

Multiple factors may influence the adherence and the efficacy of inhalation therapy including device technique, drug formulation and inhalation pattern. In addition, inhalation therapy is limited by factors such as patient's age and comorbidities. Neonates, children and geriatric patients represent patient collectives in which conventional inhalation therapy may be less effective or not feasible. The physiological specificities to be considered in infants and children are due to the process of growth and maturation. The main difficulties arise around aspects such as lip closure when using a mouthpiece, the manual power required for actuation of metered‐dose inhalers (MDIs) and the coordination of the actuation and inhalation maneuver. The physiological specificities to be accounted for in the elderly include a progressive reduction in the compliance of the chest wall, a reduction in strength of the respiratory muscles, and anatomical changes to the lung parenchyma and peripheral airways. The functional consequences in paediatric and geriatric patients are decreased peak inspiratory and expiratory airflows due to limited vital capacity.

Apart from these considerations, especially in paediatric and geriatric patients, stable chronic disease may represent another condition than acute exacerbation in respect of ability of correct device usage.

All of these factors may contribute to decreased drug delivery and reduced therapeutic efficacy as well as a possible increase in side effects.^1^

Numerous devices for inhalative drug administration have been developed.

In general, inhalation therapy devices are categorized as (1) nebulizer: compressed air jet or ultrasound nebulizer; (2) MDI: conventional pressurized MDI, breath actuated MDI; (3) dry powder inhaler (DPI); and (4) soft‐mist inhaler (SMI). Inhalation masks, mouth‐pieces, spacers, valved holding chambers (VHCs) and inhalation co‐training of caregivers represent specific technical and educative approaches to facilitate the inhalation procedure and to increase the delivery efficiency. Each approach is characterized by specific advantages and disadvantages, which may be considered in the decision process for a specific inhalation device (Table 1). Overall, inhalation therapy has to be regarded as a highly individualized principle of therapy.

**TABLE 1:** Inhaler devices: advantages and disadvantages.

Inhaler device		Advantages	Disadvantages
Small volume nebulizer	Jet nebulizer	‐ Patient coordination not required ‐ Effective with tidal breathing, breath‐hold not necessary ‐ Manual dexterity and power not necessary for inhalation ‐ Peak inspiratory flow not relevant for efficacy ‐ Good cognitive status not required ‐ Application of high doses possible ‐ Dose modification possible ‐ Combination therapies feasible ‐ Supplemental oxygen administration possible ‐ No propellants	‐ Not portable ‐ Pressurized gas source required ‐ Long preparation and treatment time ‐ Contamination possible ‐ Manual dexterity for device preparation and cleaning required ‐ Limited availability of medication with soluble formulations ‐ High cost of device/compressor ‐ Potential aerosolization of infectious particles
	Ultrasonic nebulizer	‐ Patient coordination not required ‐ Effective with tidal breathing, breath‐hold not necessary ‐ Manual dexterity and power not necessary for inhalation ‐ Peak inspiratory flow not relevant for efficacy ‐ Good cognitive status not required ‐ Application of high doses possible ‐ Dose modification possible ‐ Small dead volume ‐ Quiet ‐ Small and portable ‐ Fast delivery ‐ No drug loss during exhalation ‐ No propellants	‐ High cost of device ‐ Need for electrical power source (portable units available) ‐ Long preparation and treatment time ‐ Contamination possible ‐ Manual dexterity for device preparation and cleaning required ‐ Limited availability of medication with soluble formulations ‐ Potential drug degradation/airway irritation with some drugs ‐ Potential aerosolization of infectious particles
	Mesh nebulizer	‐ Patient coordination not required ‐ Effective with tidal breathing, breath‐hold not necessary ‐ Manual dexterity and power not necessary for inhalation ‐ Peak inspiratory flow not relevant for efficacy ‐ Good cognitive status not required ‐ Application of high doses possible ‐ Dose modification possible ‐ Small dead volume ‐ Quiet ‐ Small and portable ‐ Fast delivery ‐ No drug loss during exhalation ‐ No propellants	‐ High cost of device ‐ Need for electrical power source (portable units available) ‐ Long treatment time ‐ Contamination possible ‐ Manual dexterity for device preparation and cleaning required ‐ Limited availability of medication with soluble formulations ‐ Potential drug degradation/airway irritation with some drugs ‐ Potential aerosolization of infectious particles
Metered‐dose inhaler (MDI)	Pressurized metered‐dose inhaler (conventional)	‐ Small and portable ‐ Short treatment time ‐ Peak inspiratory flow of minor relevance for efficacy ‐ No contamination of content ‐ No preparation time ‐ High dose–dose reproducibility (dose counter in newer devices)	‐ Coordination of inhalation and actuation ‐ Requires breath‐hold ‐ Requires good cognitive status ‐ Manual power for device actuation necessary ‐ Risk of high pharyngeal deposition ‐ Potential for abuse ‐ No exact dose counting ‐ Propellants ‐ Upper dose limit
	Breath‐actuated metered‐dose inhaler	‐ No coordination of breathing and actuation necessary	‐ Range of drug content ‐ Higher costs
Supportives	Valved holding chamber, spacer (used in combination with MDI)	‐ Reduced need for patient coordination ‐ Reduced pharyngeal deposition	‐ For some patients more complex inhalation ‐ Difficulties in connecting inhaler with spacer ‐ Risk of reduced dose availability (drug deposition at spacer walls) ‐ Patient does not feel entering of medication in airways ‐ Reduced portability ‐ Additional costs
	Inhalation mask (used in combination with MDI)	‐ Reduced need for adequate lip closure	‐ Risk of reduced dose availability (drug deposition at face mask walls and skin) ‐ Increased dead space ‐ Difficulties in connecting components ‐ Additional costs
Dry‐powder inhaler (DPI)	Dry‐powder inhaler single dose devices multidose and multiunit devices power‐assisted devices	‐ Breath‐actuated ‐ Small and portable ‐ Short treatment time ‐ Reduced need for patient coordination ‐ Reduced manual power is not critical ‐ No propellants (dose counter in most devices)	‐ Requires moderate to high inspiratory flow (exception: power‐assisted devices) ‐ Requires breath‐hold ‐ Requires good cognitive status ‐ Risk of high pharyngeal deposition ‐ Some devices require manual dexterity in preparation ‐ Some units are single dose ‐ Confusing diversity of device technologies ‐ Patient does not feel entering of medication in airways
Soft‐mist inhaler (SMI)		‐ Small and portable ‐ Short treatment time ‐ Peak inspiratory flow of minor relevance for efficacy ‐ Reduced need for patient coordination ‐ No contamination of content ‐ No cleaning necessary ‐ No preparation time ‐ Less pharyngeal deposition ‐ Reduced speed of aerosol cloud ‐ No propellants	‐ Requires breath‐hold ‐ Requires good cognitive status ‐ Manual power for device actuation necessary

In the current guidelines and previously published reviews, dedicated recommendations are given for the use of different drugs adapted to specific situations.^2^, ^3^, ^4^ Increasing evidence may aid the decision‐making process in the selection of an age‐appropriate specific inhalation device in young and geriatric patients.

### 4.1 Device technologies

For drug delivery to the bronchiole of the lower respiratory tract and lungs, particle sizes of 2–5 μm (fine) or <2 μm (extra‐fine) are favoured as more peripheral deposition may be achieved. Modern inhaler devices are characterized by pulmonary deposition fractions of 40%–50% of the nominal dose, compared to fractions of 10%–15% in older devices.^5^

#### 4.1.1 Nebulizers

In nebulizers, drug solutions and suspensions are physically transformed to small aerosol droplets. Nebulizers allow the application of high dose drug formulations. Different technical solutions are used to generate drug aerosols, the most important of which are the jet and the ultrasonic nebulizer techniques. Both techniques may be applied to administer beta‐agonists, anti‐cholinergic agents and inhaled corticosteroids. In jet nebulizers, a compressor (compressed air or oxygen) is used, and fragmentation of the solution is reached by atomization producing droplets with a diameter of up to 5 μm. Ultrasonic nebulizers use a piezoelectric transductor that converts potential differences in vibration and thereby allows aerosolization of the drug solutions (in some products less effective for corticosteroids).^6^

Mesh nebulizers represent a third technical approach using a vibrating mesh disc (contraction and expansion of a vibrational element results in upward and downward movements of a domed aperture plate with tapered holes) or a vibrating horn (piezoelectric crystal vibrates at a high frequency when electrical current is applied, a transducer horn causes upward and downward movement of the mesh plate).^7^ The drug delivery is controlled by a microchip and an adaptive aerosol system that pulses the drug exclusively during the inhalation phase.

Overall, nebulizers may be indicated in persons who are either unwilling or unable to use inhaler devices. The introduction of portable units to the market made nebulizers attractive for routine use in these patients.

#### 4.1.2 MDIs and supportive devices

The central technical element is a metering valve that dispenses a predefined propellant volume including the aerosolized drug dose. Apart from propellant and drug, a surfactant substrate may be added to reduce particle agglomeration and to maintain homogenous dispersion of the drug. All components are held under high pressure in an aluminium container and are set free by decompression in the metering valve. The decompression is manually triggered by pressing the bottom of the canister against the actuator. By the power of decompression, a dispersion of aerosolized particles is ejected. In the administration forms in which the drug is not soluble in the propellant gas, the device must be shaken before usage in order to prepare a homogenous solution before applying a puff.

Pressurized MDIs represent the most frequently prescribed inhaler devices. Patients appreciate the small size, which allows the use as a pocket device. The main disadvantages are the necessity of sufficient manual power, of precise coordination of the actuation and inhalation maneuver, and the lack of dose counters in the older models.

Breath‐actuated MDIs represent another technical solution for patients with reduced coordination abilities. The inhalative flow triggers the release of the drug ensuring the coordination of inhalation and actuation. The lung deposition rate is comparable to the standard pressurized MDIs in patients with adequate coordinative abilities.^8^

The fine dispersion of the aerosolized drug may be supported by using spacers, which increase lung deposition and reduce the oropharyngeal drug deposition. This approach decreases the risk of oropharyngeal candidiasis under treatment with inhalative corticosteroids. Spacers require a certain degree of coordination between manual MDI actuation and inhalation maneuver. This problem may be solved by the use of a one‐way valve. In the nomenclature, spacers with a one‐way valve are classified as VHCs to clearly distinct between technical devices with and without a valve. VHCs ensure a unidirectional flow of the aerosolized drug out of the chamber by preventing perturbations in the spacer during tidal breathing. The positive effect of increased lung deposition is reached by accepting a slightly higher inhalation resistance in most VHCs. Comparing spacers and VHCs, there is no clear evidence that one technology is more effective than the other. The prescription of a specific supporting device should remain an individual decision process. Overall, spacers and VHCs represent simple technical approaches for patients with impaired inhalation maneuver or coordination abilities, namely in paediatric, geriatric or co‐morbid patients, the lung deposition may be increased by 30%–120%.^9^

#### 4.1.3 DPIs

In DPIs, agglomerates of drug particles or drug particles adherent to lactose carriers represent the most common drug formulations. Depending on the formulation, the device allows deagglomeration of the drug and drug release from the carrier substrates, respectively, to enable the deposition of drug particles of at most 5 μm in diameter. Single dose devices have to be separated from multidose/multiunit devices and power‐assisted devices. The latter address the fact that in progressive disease, some patients may not be able to generate an adequate PIF. The device technology uses battery‐driven impellers and vibrating piezoelectric crystals to support the dispersion of the drug particles. Apart from handihalers, where a PIF of 20 L/min is sufficient, these active (or power‐assisted) devices allow adequate lung deposition of >40% in patients with suboptimal PIF rates of about 30 L/min or less.^10^

The most important advantage of DPIs is that no coordination of actuation and inhalation is necessary. However, a limitation is the obligatory PIF rate, especially in children under the age of 5 years and adults with severely impaired lung function. The PIF required for adequate drug liberation differs substantially between DPI devices. The resistance is low in Breezhaler; medium‐low in Accuhaler, Diskhaler, Diskus and Ellipta; medium in Clickhaler, Genuair/Pressair, Spiromax/RespiClick/Digihaler and Turbuhaler (Symbicort); medium‐high in Easyhaler (combination therapy), Nexthaler, Turbuhaler (Pulmicort) and Twisthaler; high in Easyhaler (monotherapy) and Handihaler.^11^ In the presence of reduced inspiratory flow, power‐assisted devices represent an attractive but relatively cost‐intensive solution. An advantage may be the environmental impact as DPIs are free of propellants and reusable devices may reduce plastic waste.

Multiple different device technologies of DPIs have been developed and introduced to the market by promoting technical specificities. The multitude of devices may lead to confusion and the risk of lacking skills of usage education in patients as well as in physicians.

The usage of some DPI devices may be limited as impaired manual dexterity may result in handling problems with essential steps (opening of medication blisters, insertion of capsules, manual device activation steps to allow drug release).

In addition, there is no clear evidence that certain products are per se superior compared to the others. Consequently, the guidelines do not recommend certain products but advise to practise shared decision making together with the patients and caregivers. Furthermore, the number of different device types should be minimized and device types should not be switched without a clear indication.

#### 4.1.4 SMI

The SMI is a propellant‐free multidose inhaler where a metered dose of drug solution is set free by a uniblock nozzle. The nozzle generates soft mist of aerosol. In pressurized MDIs, the mean velocity of the generated aerosol at a 10 cm distance is about 2.0–8.4 m/sec with a mean duration of aerosol liberation of 0.15–0.36 s. Compared to this, the mean velocity of the generated aerosol at a 10 cm distance from the SMI nozzle is 0.8 m/sec, the mean duration of aerosol liberation is 1.5 s.^12^ In conclusion, SMIs are characterized by small particle sizes, higher lung deposition, reduced ejection speed and longer aerosol liberation, which may be attractive for use in patients with reduced abilities in actuation/inhalation coordination.

### 4.2 Limitations in inhaler usage

None of the previously published pooled meta‐analyses of these devices showed a significant difference in any efficacy outcome nor in any patient group for each of the clinical settings that were investigated.^1^, ^13^ Each of the delivery devices provided similar outcomes in patients using the correct technique of inhalation.^1^ However, it has to be considered that the majority of comparative studies were designed to show the equivalence among different devices in highly selected and trained patients.^5^ Apart from highly selected trial populations, limitations in inhaler usage may be a relevant problem in the real‐life scenario.

Sulaiman et al. demonstrated that the main errors in inhaler use relate to problems with inspiratory flow, inhalation duration, coordination, dose preparation, exhalation maneuver prior to inhalation and breath‐hold following inhalation maneuver.^14^

### 4.3 Age‐specific limitations

The GINA and GOLD recommendations point out that up to 70%–80% of patients are unable to use their inhalers correctly and are unaware of the fact that their mode of application is ineffective. Known risk factors for inappropriate inhaler usage are older age, use of multiple devices and lack of education in inhaler usage.^15^ In their meta‐analysis of asthma patients in 14 studies, Barbara et al. found evidence that, despite a heterogenous dataset, increasing age is associated with increasing proportions of incorrect MDI and DPI users.^16^ Usmani et al. analysed 41 studies reporting data on critical MDI and DPI user errors in asthma and COPD patients.^17^ They found age, education status, previous inhaler instruction, comorbidities and socioeconomic status to be associated with frequently worse handling. Thirty‐three of the analysed studies examined the effect of patient age (29 in adults, four in paediatric patients). Only 12/33 found age to be associated with worsening frequency of inhaler errors. More recent studies could demonstrate that device usage errors are more common in older patients.^18^, ^19^

In one paediatric study, it was analysed whether there were differences in the usage of a spacer in combination with a mouthpiece versus a spacer combined with a face mask. Interestingly, it could be shown that more critical errors occurred in the spacer‐mouthpiece group. It was demonstrated that inhaler technique errors were most prominent in the adolescent population, possibly coinciding with the process of transitioning to a mouthpiece and more independence in medication administration.^20^

#### 4.3.1 Paediatric patients—problems and solutions

Defining strategies for inhalation therapy in paediatric patients is difficult. In theory, most devices may be applicable for use in children. However, many inhaled drugs are used off‐label in paediatric patients, which is either due to the use for a different indication in a younger age group or combined with an individual support device. Evidence in this field is growing but sparse.^21^ In addition, the optimal size of aerosol particles has not yet been determined for certain situations such as when artificial airways (e.g., spacers, VHCs) are used or when the application is performed via the oronasal orifices. The evidence for transnasal aerosol inhalation therapy in infants and children is limited, and the efficacy in clinical routine remains unclear.^22^

According to the GINA recommendations, a pressurized MDI with a VHC (with or without face mask, depending on the child's age), represents the preferred delivery system for paediatric asthma patients. However, the dose delivered by the VHC may vary between the different models. Models with documented efficacy in children should be given preference. As young children are completely reliant on tidal breathing during inhalation therapy, the time for whole dose consumption may vary depending on the tidal volume of the child and the chamber's dead space and volume. Lower volumes may be preferred in young children. However, the data is heterogeneous, as some studies did not detect any differences in bronchodilator responsiveness in relation to spacer device selection.^23^ VHCs made of antistatic materials should be preferred in order to reduce drug loss caused by drug attraction due to static charge on some plastic spacers.

Nebulizers are reserved for the children who are not able to use spacers adequately. This inability may be due to age, maturity, comorbidities or acute psychological stress in the situation of emergency. There is little evidence regarding the necessary skills or the optimal age for transitioning children from the mask‐plus‐VHC concept to a mouthpiece‐plus‐VHC concept.^24^ Herbes et al. analysed MDI therapy with a VHC and face mask in 117 low‐risk newborns and showed that only 37% were able to generate a sufficient negative pressure to open the chamber valve.^25^

Iramain et al. demonstrated in 103 children (aged 2–14 years) who were admitted to the emergency room with a severe acute exacerbation of asthma, that administration of salbutamol/ipratropium by MDI with VHC and face mask was more effective than a nebulizer therapy.^26^ Snider et al. analysed 890 patients (aged 2–17 years) with mild to moderate asthma exacerbations and demonstrated the non‐inferiority of MDI albuterol therapy compared to breath‐actuated albuterol nebulizer therapy.^27^

#### 4.3.2 Comorbid and geriatric patients—problems and solutions

In general, in elderly patients, coordination problems and cognitive impairment may play a relevant role in inhaler misuse, though the problems may be resolved by focused training.^28^, ^29^

It is important to estimate the patient's PIF capacity when the initiation of DPI therapy is being considered. In COPD patients, it has been shown that using different DPI devices leads to changes in PIF and thereby to potential variations in drug deposition.^30^ It may be challenging to estimate the PIF in clinical routine to predict the potential benefit from DPI devices. Disease severity does not per se predict the feasibility of DPI therapy as peak expiratory flow may not correlate with PIF. Spirometry delivers PIF values but is not available in all patients. Maximum airflow generated during inhalation in litres per minute against the simulated resistance of a DPI (PIFr) is regarded as a predictor of delivery efficiency of DPI devices.^11^ By this, PIF metres with adjustable resistance may aid with device selection. Handgrip strength measurement represents a simple clinical test potentially correlating with the PIF. Frohnhofen and Hagen could demonstrate that results from handgrip strength measurements could be a predictor for a successful DPI therapy in geriatric patients with COPD.^31^ Recently, the concepts of breath‐actuated MDIs and SMIs have been established, which show advantages especially in aged or arthritis patients with problems in coordination of actuation and inhalation. These devices can be actuated by low airflow rates, which makes the technique attractive for patients at all severities of disease.

Shirmanesh et al. demonstrated a reduced ability of rheumatoid arthritis patients to manually complete all steps to operate their device.^32^ Similar problems are observed in patients with visual impairments or neurologic disorders like Parkinson's disease and status post stroke. Patients who remain unable to effectively use handheld inhalers despite instruction should be considered for an alternative approach. In case that personal assistance by educated family members or caregivers is available, these resources should be used to allow MDI therapy. Optionally, the therapy may be supported by additional tools like face mask, spacer or VHC. This approach is highly effective and time‐sparing. Therefore, it should be favoured over nebulizer use, which would remain a kind of a last resort. However, nebulizers should be considered in all patients with cognitive impairment, inadequate manual dexterity or manual weakness when personal assistance is not sufficiently available.

In theory, the inhalation of extra‐fine particles may lead to improved topical drug effects in the smaller airways. However, in the study of Price et al., it was demonstrated that there were no relevant differences between the effects of fine versus extra‐fine particles. Interestingly, the results of the elderly patients (aged 61–80 years, 37% of all patients) were similar to those of the whole cohort.^33^

### 4.4 Improving efficacy of the established inhalator therapy

Overall, there is only limited evidence on the effect of interventions to improve the inhaler technique.^34^ The critical population at risk (paediatric, geriatric and comorbid patients) is not well studied. Most of the data discussed above are derived from retrospective analysis or small prospective studies in a heterogenous population. The critical population at risk represents only a subgroup in some studies. Only in few studies, the population at risk was predefined as primary endpoint. By this, the reported data are hypothesis‐generating but should be substantiated by further prospective studies.

Recent data could suggest that there is a need for focused surveillance in the transition periods from infancy to childhood to adolescence and later to senescence.^20^ These transition steps represent cornerstones of disease management where devices and supporting tools may be adjusted according to individual conditions. In these vulnerable situations, a concise education has to ensure correct device utilization in order to maintain maximal lung deposition of the drug.

In general, confusing technical specificities of the available inhalation devices and lack of skills in device usage among both patients and health care providers, may contribute to insufficient therapy benefit.

There are, however, strategies to ensure an appropriate inhaler usage. Dedicated patient education by skilled personnel should be followed by repetitive demonstration of application by the patient at the follow‐up visits and correction of detected mistakes in device usage. In geriatric patients, repetitive and intensive device training significantly reduced handling mistakes.^29^ Where possible, a single device technology should be implemented, as this concept increases adherence and disease control.^1^

### 4.5 The decision‐making process for an inhaler device

In conclusion, age, cognitive status, visual acuity, manual dexterity, manual strength and coordination abilities may be as important factors as the disease severity for the individual decision‐making process for optimal inhalation therapy. Comorbidities, such as cardiovascular and generalized arteriosclerosis, chronic renal failure, osteoporosis, obesity, polyneuropathy, arthritis, cognitive, visual and auditory impairment, may influence the treatment choices. Furthermore, polypharmacy and age‐related changes in pharmacokinetics and pharmacodynamics should be considered to reduce adverse drug effects, drug–drug or drug–disease interactions and therapy incompliance.

### 4.6 Reasonable strategy

The modern concepts of inhalation therapy imply the need for a highly individualized approach with close follow‐up and monitoring.^35^

For infants and children, a strategy that respects maturity and psychomotor skills may be reasonable, taking into account the acceptance of the device by the caregiver. In elderly patients, the concept should be adapted according to the limitations caused by cognitive impairments or comorbidities. A proposal for an algorithm for the individual decision‐making process is given in Table 2.

**TABLE 2:** Proposed algorithm for the decision‐making process for inhaler device prescription (assuming that all formulations are available in all devices).

Age (years)	0–2	3–4	5–6	>6									
				Coordination of actuation and inhalation	+	+	+	+	−	−	−	−	+/−
				manual dexterity and power	+	+	−	−	+	+	−	−	+/−
				peak inspiratory flow >30 L/min	+	−	+	−	+	−	+	−	+/−
				cognitive status	+	+	+	+	+	+	+	+	−
Recommended device	MDI with spacer/VHC and mask with assistance for actuating (#); small‐volume nebulizer with mask	MDI with spacer/VHC and mask or mouthpiece with assistance for actuating (#); small‐volume nebulizer with mask or mouthpiece	MDI with spacer/VHC and mouthpiece; (DPI); nebulizer with mouthpiece		MDI, DPI, SMI	MDI, SMI	DPI, MDI, SMI with assistance for preparing and actuating (#); nebulizer with mouthpiece	MDI, SMI with assistance for preparing and actuating (#); nebulizer with mouthpiece	DPI; MDI with spacer/VHC and mouthpiece; SMI breath‐actuated MDI	MDI with spacer/VHC and mouthpiece; SMI breath‐actuated MDI	MDI with spacer/VHC and mouthpiece, SMI with assistance for preparing and actuating (#); DPI with assistance for preparing (#); nebulizer with mouthpiece	MDI with spacer/VHC and mouthpiece, SMI with assistance for preparing and actuating (#); nebulizer with mouthpiece	MDI with spacer/VHC and mask or mouthpiece, SMI with assistance for preparing and actuating (#); nebulizer with mask or mouthpiece

Abbreviations: +, criteria fulfilled; −, criteria not fulfilled; +/−, criteria fulfilled or not fulfilled; #, assistance in preparing and actuating the inhalation device by educated parents (in children) or caregivers (in comorbid or geriatric patients); DPI, dry powder inhaler; MDI, metered‐dose inhaler; SMI, soft‐mist inhaler; VHC, valved holding chamber.

Pressurized MDI may be suitable for patients who fulfill all cognitive, coordinative and manual power requirements. Breath‐actuated MDIs, SMIs, VHCs, spacers and face masks may be suitable for patients with mild to moderate impairments of one or more of these variables. In these cases, available family members and caregivers should be trained to assist with the inhalation therapy.

DPIs may be applicable, irrespective of coordination skills but are reserved for patients with a sufficient PIF and good cognitive and manual abilities.

Nebulizers represent a reasonable solution for patients who are unsuitable for handheld devices, especially for the very young, elderly and acutely ill patients where personal assistance of family members or caregivers is not available.

The preferences of the patient should be integrated into the decision‐making process. Where possible, a single device technology should be implemented, as this concept increases adherence and disease control. Mixed devices should be avoided.

Finally, upon initiation of a specific inhalation therapy, close monitoring is essential.

### 4.7 Unanswered questions and future directions

Inhalation strategies are often ineffective in very young and elderly patients. Evidence in this field is still growing but sparse. More studies that address the endpoints symptom relief, patient satisfaction with the therapeutic concept and adherence are needed. In paediatric patients, real‐life data do not reflect data from large clinical trials; off‐label strategies are common. More data is needed to prove the benefit of these approaches. The demographic change leads to an increase of geriatric patients in health care facilities. However, these patients are underrepresented in clinical phase 3 trials.

Limited delivery efficiency is still a major issue of current device technologies. Delivery may be affected by characteristics of the devices and also by patient's cognitive resources, coordination between inhalation and actuation, inspiratory flow and breathing pattern. Recent developments aimed to generate particle sizes with better penetration to the targeted sites and to allow easier activation of the device with inspiratory flow. Computer chips were used to develop smart delivery devices, which provide feedback to patients; a strong confirmation of a favourable cost/benefit ratio with this approach is pending.^35^ In everyday practice, smart technologies may not be applicable with the population, in which drug administration is critical (children, geriatric and comorbid patients). Increasing the ease of use by including fewer steps in using the device and reaching a better portability are aims that still need to be addressed. These considerations demonstrate that addressing single technical issues may not be goal‐oriented and may not overcome the general problem of limited drug delivery and adherence.

Innovative approaches may help to overcome the problem of low lung deposition of the inhaled drug. First data are reported from new formulations like nanoparticles and several small molecules. A new research field is the area of targeted carrier systems (e.g., polymeric particulate carriers, lipid based carriers or viral DNA/RNA vectors). These carriers may be used as vehicles for conventional synthetic drugs and also for nanoparticles, peptides or nucleic acids.^36^ The clinical applicability is still unclear. Nevertheless, the results from preclinical studies are encouraging, and data from clinical trials are awaited.

## 5 CONCLUSIONS

Inhalation therapy has to be regarded as a highly individualized concept of therapy. Age, maturity and comorbidities should be considered when choosing a specific inhalation device. Standardized algorithms may support this decision process. A close monitoring is essential as it may detect handling mistakes and the need to modify the device concept. In the future, novel approaches may increase the therapeutic effect by overcoming the problem of limited drug deposition in the small airways.

## AUTHOR CONTRIBUTIONS

**Lars Hagmeyer**: Substantial contributions to conception and design; analysis and interpretation of available data; drafting the article; and finalizing the version to be published taking responsibility for the integrity of the work as a whole, from inception to published article. **Silke van Koningsbruggen‐Rietschel**: Substantial contributions to conception and design; analysis and interpretation of available data; drafting the article and finalizing the version to be published taking responsibility for the integrity of the work as a whole, from inception to published article. **Ernst Rietschel**: Substantial contributions to conception; analysis and interpretation of available data; revising the article critically for important intellectual content; final approval of the version to be published. **Sandhya Matthes**: Analysis and interpretation of available data; revising the article critically for important intellectual content; final approval of the version to be published. **Winfried Randerath**: Substantial contributions to conception; analysis and interpretation of available data; drafting the article; and finalizing the version to be published.

## CONFLICT OF INTEREST STATEMENT

Lars Hagmeyer reports grants and personal fees from Roche, grants and personal fees from Böhringer Ingelheim, personal fees from Lilly, grants from MSD and Astra Zeneca, outside the submitted work.

Silke van Koningsbruggen‐Rietschel states grants from HORIZON2020 OligoG pivotal Cystic Fibrosis and honoraria from Deutsches Zentrum für Infektionsforschung, Antabio, Boehringer.

Ernst Rietschel states participation in advisory board of Mylan.

Sandhya Matthes states that there is no conflict of interest.

Winfried Randerath reports grants and personal fees from Böhringer Ingelheim and Berlin Chemie. Winfried Randerath is Associate Editor of the journal ‘Respiration’.

## ETHICS STATEMENT

No ethics approval was needed, no human subjects were involved in this review paper, and no consent to participate and publish was needed.

## Data Availability

Data sharing is not applicable to this article as no new data were created or analyzed in this study.
